# *Mycoplasma pneumoniae* CARDS Toxin Is Internalized via Clathrin-Mediated Endocytosis

**DOI:** 10.1371/journal.pone.0062706

**Published:** 2013-05-07

**Authors:** Manickam Krishnan, T. R. Kannan, Joel B. Baseman

**Affiliations:** Department of Microbiology and Immunology, The University of Texas Health Science Center at San Antonio, San Antonio, Texas, United States of America; University of Birmingham, United Kingdom

## Abstract

Bacterial toxins possess specific mechanisms of binding and uptake by mammalian cells. *Mycoplasma pneumoniae* CARDS (Community Acquired Respiratory Distress Syndrome) toxin is a 68 kDa protein, which demonstrates high binding affinity to human surfactant protein-A and exhibits specific biological activities including mono-ADP ribosylation and vacuolization. These properties lead to inflammatory processes in the airway and a range of cytopathologies including ciliostasis, loss of tissue integrity and injury, and cell death. However, the process by which CARDS toxin enters target cells is unknown. In this study, we show that CARDS toxin binds to mammalian cell surfaces and is internalized rapidly in a dose and time-dependent manner using a clathrin-mediated pathway, as indicated by inhibition of toxin internalization by monodansylcadaverine but not by methyl-β-cyclodextrin or filipin. Furthermore, the internalization of CARDS toxin was markedly inhibited in clathrin-depleted cells.

## Introduction

*Mycoplasma pneumoniae* is an atypical bacterium that causes respiratory illnesses in humans, including pharyngitis, tracheobronchitis, and community-acquired pneumonia [1], [2]. It has also been directly linked to reactive airway disease, asthma and extrapulmonary pathologies [3], [4]. *M. pneumoniae* has been detected in the airway samples of up to 25% of asthmatics experiencing acute exacerbations [5], [6].

The interaction of *M. pneumoniae* with the airway epithelium results in significant cytopathology both in organ culture and *in vivo* [7], [8], [9]. Previously, the cytopathology induced by *M. pneumoniae* infection was linked in part to hydrogen peroxide and superoxide radicals generated by mycoplasma metabolism [7], [10]. Recently, we identified a novel ADP-ribosylating and vacuolating cytotoxin of *M. pneumoniae* designated Community Acquired Respiratory Distress Syndrome (CARDS) toxin capable of inducing cytopathology both *in vitro* and *in vivo* that reproduces the infectious process [11], [12].

The amino terminal region of CARDS toxin shares 27% identity with pertussis toxin S1 subunit (PTX-S1) and retains the necessary motif and essential amino acids for ADP ribosylation of host proteins [13]. In addition, CARDS toxin induces vacuolization in mammalian cell lines, tracheal organ cultures and *in vivo* [11], [12]. Other observations indicate that CARDS toxin uses unique pathways to generate vacuoles that are distinct from *Helicobacter pylori* vacuolating cytotoxin-VacA [14].

*M. pneumoniae* poorly expresses CARDS toxin during *in vitro* growth but dramatically increases synthesis *in vivo* [15]. Using specific CARDS toxin assays, we detected and co-localized *M. pneumoniae* and CARDS toxin in biological fluids of infected animals and human tissue samples [16], [17], [18]. Also, we observed dramatic seroconversion to CARDS toxin in *M. pneumoniae*-associated pneumonia patients, further indicating that this toxin is synthesized *in vivo* and possesses highly immunogenic epitopes [11].

Bacterial protein toxins act at cell surfaces or targets inside susceptible cells [19]. ADP-ribosylating bacterial toxins modify intracellular sites of action [20], which requires their traversing host cell membranes. Since recombinant CARDS (rCARDS) toxin alone elicits histopathology similar to *M. pneumoniae* infection, including the characteristic ciliostasis, cytoplasmic swelling and vacuolization, nuclear fragmentation, extensive inflammation, and tissue pathologies [11], [12], we analyzed its binding and internalization in different mammalian cell lines. We also examined the endocytic process that mediates rCARDS toxin internalization using biotin-labeled rCARDS toxin, pharmacological reagents, and genetic approaches. Data show that binding and internalization of CARDS toxin are facilitated by clathrin-mediated pathways.

## Results

### rCARDS Toxin Binds and is Internalized by Different Cell Types

**Figure 1:**
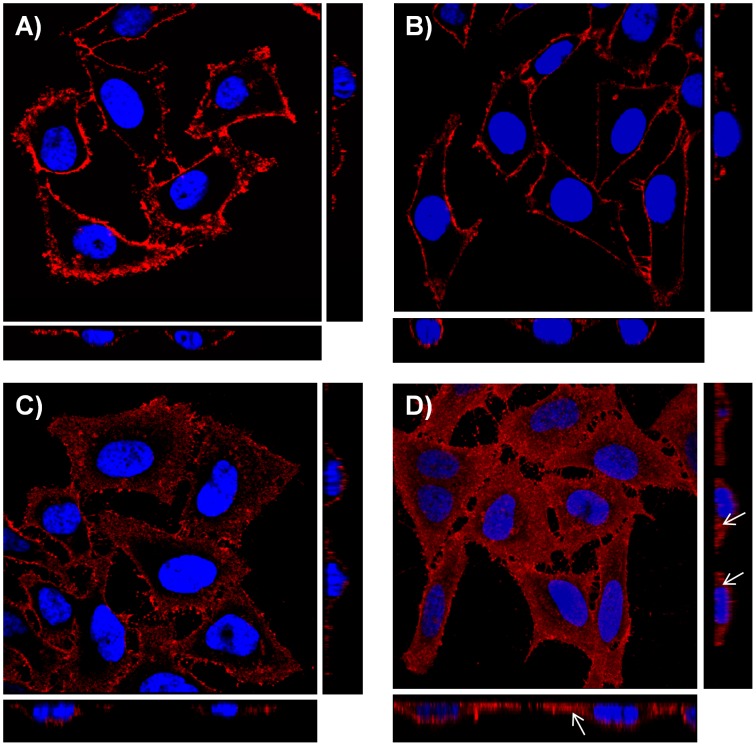
Binding and internalization of rCARDS toxin in HeLa cells. A) rCARDS toxin localizes to HeLa cell surfaces. HeLa cell monolayers were treated with 10 µg/ml of rCARDS toxin for 1 h at 4°C, stained with rabbit polyclonal antibodies reactive against CARDS toxin (1∶1000) and counterstained with goat anti-rabbit IgG conjugated Alexa Fluor-633 antibodies (red). Cell nuclei were stained with DAPI (4′,6-Diamidino-2-Phenylindole, Dihydrochloride). Z series at 0.5 micrometer sections were obtained by combining a series of x-y scans taken along the z axis. B-D) rCARDS toxin internalization is time dependent. HeLa cells were treated with 10 µg/ml of rCARDS toxin for B) 15 min; C) 30 min; and D) 1 h at 37°C. Cells were processed as described above. White arrows point to internalized rCARDS toxin based upon horizontal and vertical z projections.

Binding of rCARDS toxin to cell surfaces was determined by incubating HeLa cells with toxin (10 µg/ml) at 4°C for 30 min. rCARDS toxin was visualized as intense red fluorescence on the apical surfaces of cells using optical confocal planes; at this temperature cross sectional *z* series views did not detect toxin internalization (Fig. 1A). However, at 37°C, rCARDS toxin localized transiently to the cell surface, followed by rapid internalization as indicated by cytoplasmic punctate red fluorescence (Fig. 1B–D). Optical cross sections confirmed a time-dependent increase in cytoplasmic-associated rCARDS toxin, suggesting receptor-mediated endocytosis.

Human and other mammalian cells were analyzed to confirm the binding and internalization of rCARDS toxin. As shown by immunofluorescence confocal laser scanning microscopy, rCARDS toxin binds to and is internalized by all analyzed cell types, with distribution throughout the cytoplasm visualized by punctate red fluorescence within 1 h (Fig. S1). These data suggest that rCARDS toxin utilizes common or parallel entry pathways.

### rCARDS Toxin Binding is Dose and Time Dependent

**Figure 2:**
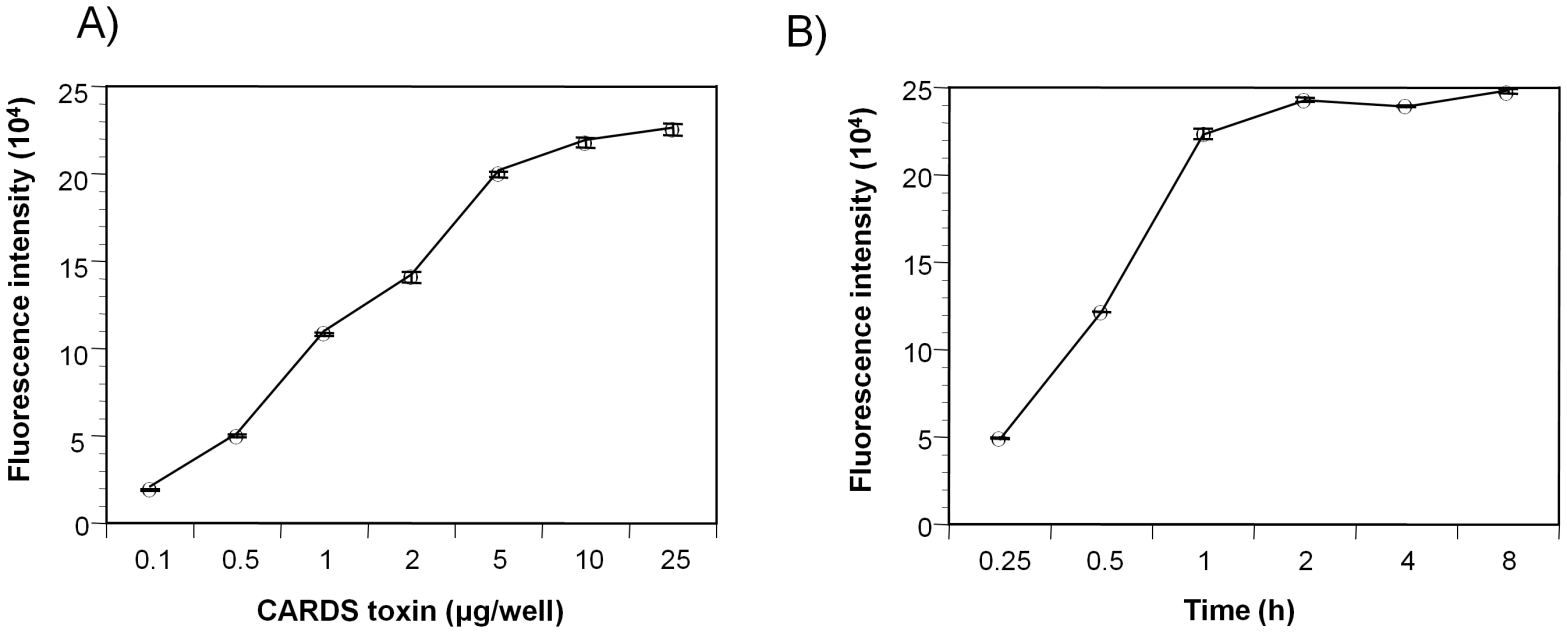
Binding kinetics of fluorescence labeled rCARDS toxin. **(**A) rCARDS toxin binding to HeLa cells is concentration dependent. HeLa cells were treated with different concentrations (0.1 to 25 µg/well) of DyLight 649 fluorescence labeled rCARDS toxin for 1 h at 4°C. Cell monolayers were thoroughly washed with cold PBS buffer, and toxin binding was measured using fluorometer. (B) rCARDS toxin binding to HeLa cells is time dependent. HeLa cells were treated with 10 µg of DyLight 649 fluorescence-labeled rCARDS toxin for 15 min to 8 h at 4°C, and binding was analyzed as described above.

We assessed cell binding of rCARDS toxin labeled with Dylight-649 fluorescence dye (DL-CARDS toxin). DL-CARDS toxin (0.1 to 25****µg/ml) bound to HeLa cells in a dose-dependent manner, and binding appeared to level off at ∼10 µg at 4°C (Fig. 2A). To examine time-dependent saturation, HeLa cells were treated with 10 µg/ml of DL-CARDS toxin at 4°C and monitored at different intervals; peak binding was achieved between 1 and 2 h (Fig. 2B). To further analyze the specificity of rCARDS toxin binding, competitive assays were performed with DL-CARDS toxin in the presence of 10-fold excess unlabeled toxin. More than 90% inhibition of the DL-CARDS toxin was observed, indicating direct competition between unlabeled and DL-CARDS toxin (data not shown).

**Figure 3:**
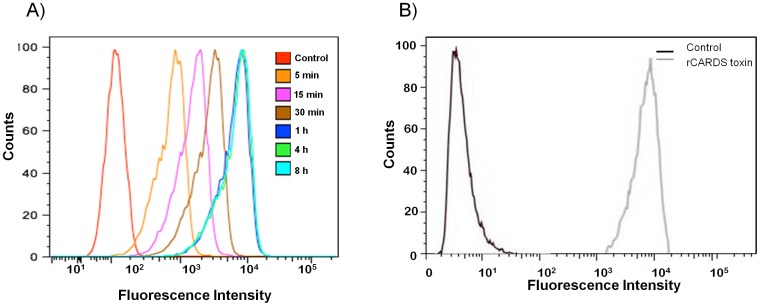
FACS analysis of rCARDS toxin association with HeLa cells. A) HeLa cells were treated with 10 µg/ml of pacific blue-A fluorescence dye-coupled toxin (PBA-CARDS) at different time intervals (5 min to 8 h) at 4°C, washed and quantified by FACS. Cells treated with carrier solution served as controls. B) HeLa cells were treated with 10 µg/ml of rCARDS toxin for 30 min at 4°C, washed and incubated with rabbit polyclonal anti-CARDS toxin antibodies (1∶1000) and counterstained with goat anti-rabbit IgG-conjugated with Alexa Fluor 488 (1∶500) followed by FACS. The control shows HeLa cells with no toxin treatment but stained with primary and secondary antibodies.

To further study saturation binding dynamics, HeLa cells were treated with 10 µg/ml of pacific blue-A labeled CARDS (PBA-CARDS) toxin at 4°C, and toxin binding was examined by flow cytometry over time (5 min to 8 h). Binding of PBA-CARDS toxin increased with incubation time and reached maximum saturation at 1 h (Fig. 3A) consistent with Fig. 2. In parallel, we used flow cytometry to monitor the extent to which unlabeled rCARDS toxin bound to HeLa cell populations. After 1 h incubation and using rabbit polyclonal anti-CARDS toxin antibodies and secondary Alexa Fluor 488-labeled anti-rabbit antibody, we detected 97.8% of cells bound rCARDS toxin (Fig. 3B).

### rCARDS Toxin is Internalized by HeLa Cells

**Figure 4:**
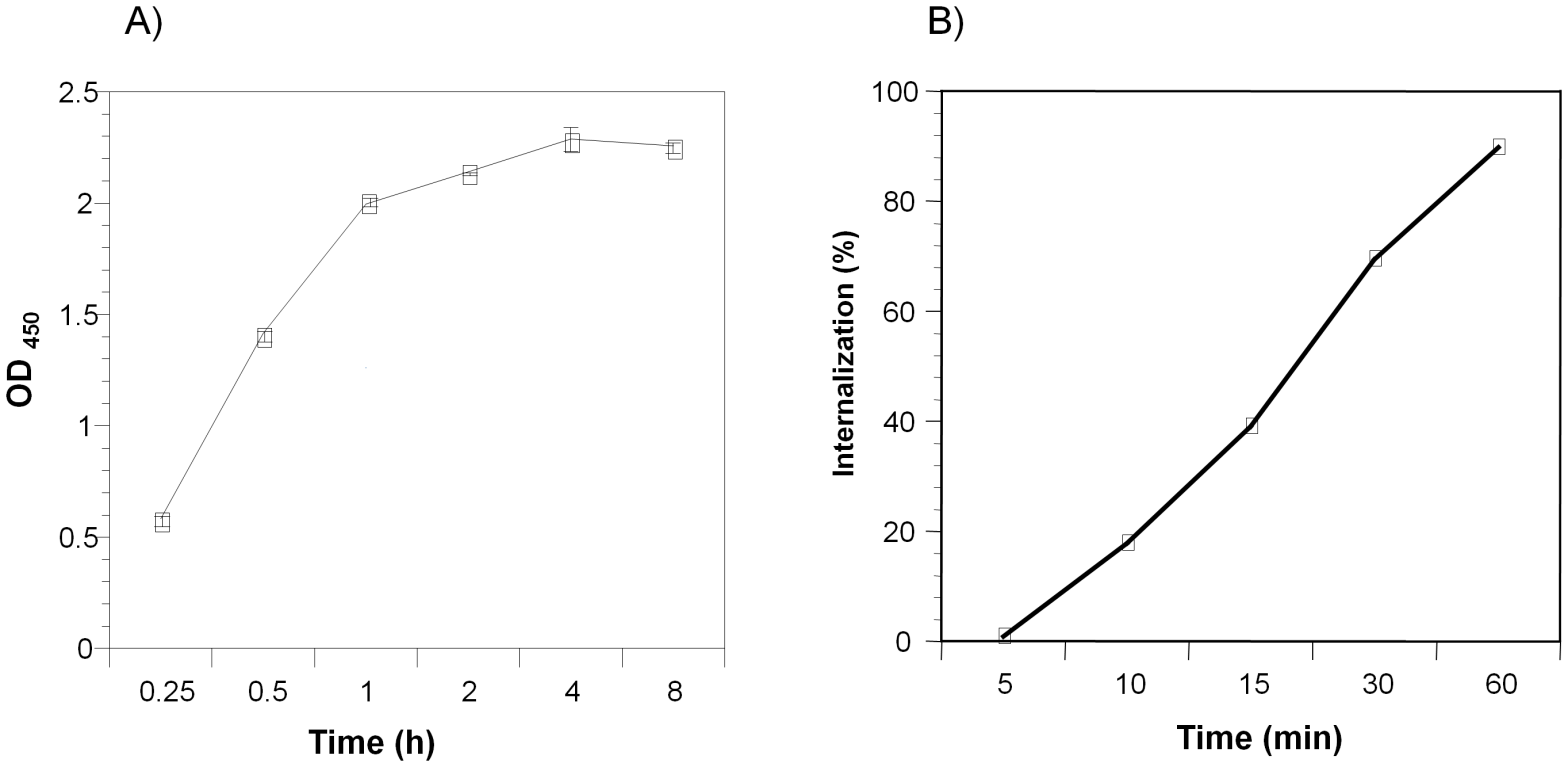
Binding and internalization of biotin-labeled rCARDS toxin. (A) Biotin-labeled rCARDS toxin binding to HeLa cells is time-dependent. HeLa cells were treated with 10 µg/ml of biotin-labeled rCARDS toxin for 15 min to 8 h at 4°C. After removing unbound toxin by washing, we analyzed binding using horseradish peroxidase-conjugated streptavidin. Cell-bound rCARDS toxin was measured at 450 nm using ELISA reader as described in Materials and Methods. (B) Internalization of rCARDS toxin in HeLa cells. HeLa cells were treated with 10 µg/ml of biotin-labeled rCARDS toxin for 30 min at 4°C and then incubated at 37°C for the indicated times after removing unbound toxin by washing. At specific time intervals, cells were fixed and surface-bound biotin was removed with MESNA. Internalized biotin-associated rCARDS toxin was quantified as described in Materials and Methods. Data are means ±SD of triplicate samples.

Internalization of rCARDS toxin was assessed using rCARDS toxin labeled with biotin (Biotin-CARDS). As observed with unlabeled or PBA-CARDS toxin, Biotin-CARDS toxin bound to HeLa cells in a similar dose-dependent manner (data not shown) and binding saturation was reached at ∼1 h (Fig. 4A). For internalization studies, 10 µg/ml Biotin-CARDS toxin was added to HeLa or A549 cells, and toxin internalization was detected at specific time points after removing biotin from surface-bound toxin, using 2-mercaptoethanesulfonic acid (MESNA) followed by cell permeabilization. The results indicated that rCARDS toxin was internalized in a time-dependent manner **(** Fig. 4B).

### Clathrin-mediated Endocytic Mechanisms are Implicated in Internalization of rCARDS Toxin

Toxins are endocytosed through clathrin-dependent [21], [22], [23] and/or independent vesicles including the caveolin-mediated mechanisms [24], [25], [26]. In order to identify the endocytic pathway(s) exploited by CARDS toxin, three different drugs that arrest caveolin- and clathrin-endocytic pathways were used. Filipin is an antifungal antibiotic that prevents caveolae vesicle formation, and filipin pretreatment of HeLa cells permitted CARDS toxin internalization (Table 1). As a positive control, we monitored cholera toxin entry, which is known to utilize caveolin-dependent endocytic pathways, and filipin dramatically reduced its internalization (Fig. S2). Monodansylcadaverine (MDC) prevents clathrin-dependent endocytosis, and pretreatment of HeLa cells with MDC blocked rCARDS toxin as well as transferrin (Tf) entry inside the cells (Table 1 and Fig. S2); Tf is known to enter cells by clathrin-mediated endocytosis. Methyl-β-cyclodextrin (MβCD) depletes cholesterol and affects clathrin-independent pathways. Pre-treatment of HeLa cells with MβCD did not block CARDS toxin or Tf internalization but reduced cholera toxin internalization (Table 1; Fig. S2). These results implicated clathrin-mediated endocytosis as a mediator of CARDS toxin uptake.

**Table 1:** Effects of endocytic pathway inhibitors on cell entry of rCARDS toxin.

Inhibitory compound	Toxin/Protein	Cell entry
**Monodansyl cadaverine** Blocks clathrin-dependent endocytosis	CARDS toxin	**−**
	Transferrin (Positive control)	**−**
	Cholera toxin (Negative control)	**+**
**Methyl-β-cyclodextrin** Blocks caveolin-dependent endocytosis	CARDS toxin	**+**
	Transferrin (Negative control)	**+**
	Cholera toxin (Positive control)	**−**
**Filipin** Blocks caveolin-dependent endocytosis	CARDS toxin	**+**
	Transferrin (Negative control)	**+**
	Cholera toxin (Positive control)	**−**

HeLa cells were pre-treated with 5 mM of Methyl-β-cyclodextrin, 100 µM of Monodansylcadaverine, or 1 µg Filipin for 30 min at 37°C. Subsequently, rCARDS toxin or transferrin or cholera toxin was added individually. Cell entry of these molecules was analyzed by confocal laser scanning immunofluorescence microscopy.

**Figure 5:**
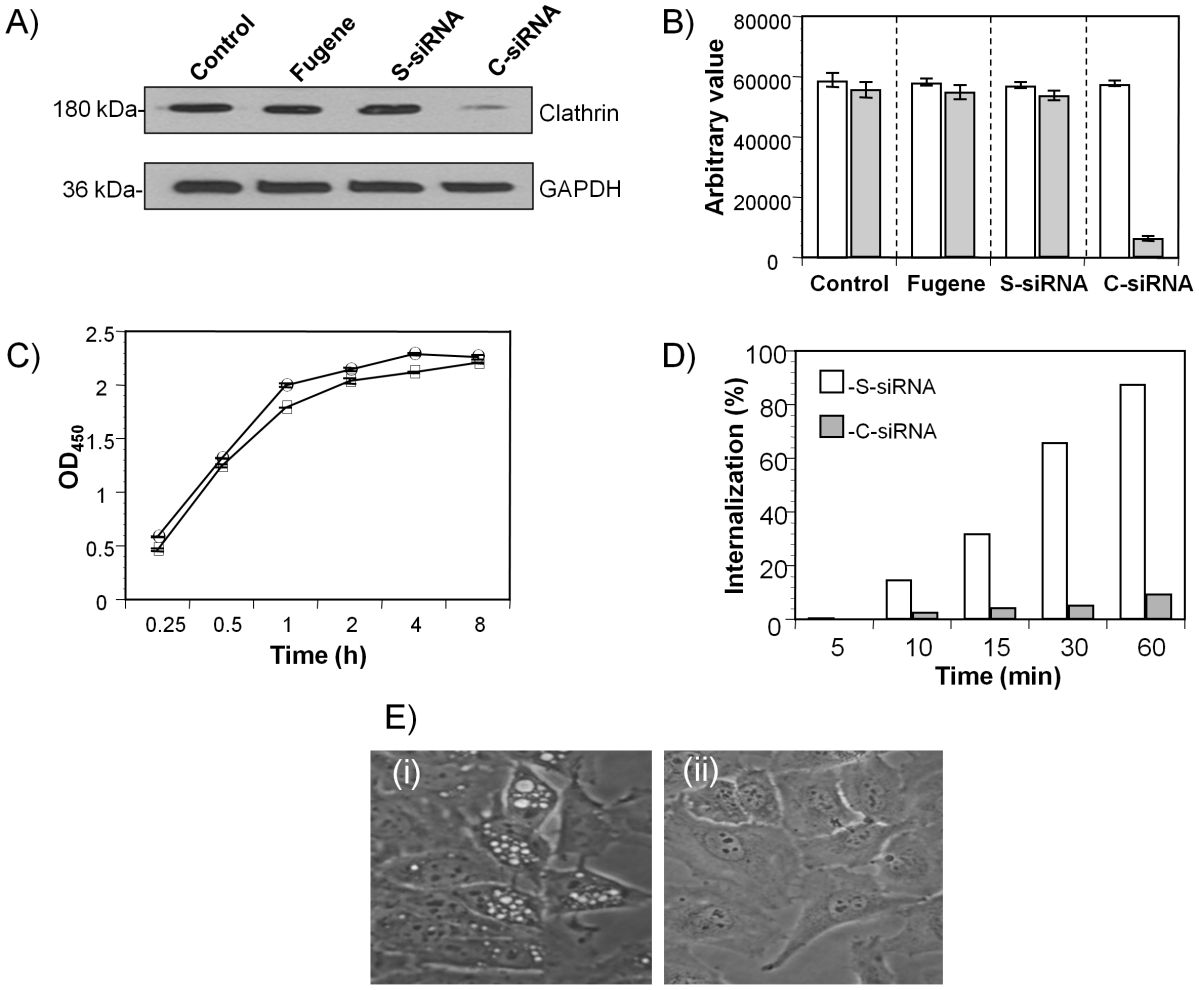
Clathrin-rCARDS toxin association. **(**A) Small interfering RNA (siRNA) mediated gene silencing of clathrin heavy chain. HeLa cells were transfected with clathrin heavy chain targeted siRNA (C-siRNA) or scrambled siRNA (S-siRNA) as described in Materials and Methods. Twenty-four h after the second siRNA transfection, cells were lysed and clathrin expression was analyzed using anti-clathrin (1∶500) or anti-GAPDH (1∶2000) antibodies as detailed in Materials and Methods and described in this legend. (B) Quantification of clathrin heavy chain expression in C-siRNA- and S-siRNA-treated HeLa cells. Clathrin and GAPDH band intensities were estimated using KODAK 1D Image analysis software, and results are from three independent experiments. (C) Binding of rCARDS toxin in clathrin-depleted cells. S-siRNA-treated (open square) and C-siRNA-treated (open circle) HeLa cells were incubated with 10 µg/ml of rCARDS toxin for 1 h at 4°C, and toxin binding was analyzed as described in Materials and Methods. Data are means ±SD values from two triplicate experiments. (D) Internalization of rCARDS toxin in clathrin-depleted HeLa cells. S-siRNA-treated (white bar) and C-siRNA-treated (gray bar) HeLa cells were incubated with 10 µg/ml of Biotin-CARDS toxin for 1 h at 4°C and shifted to 37°C for 1 h. After removing surface-bound biotin with MESNA, internalized rCARDS toxin was measured as described in Materials and Methods. (E) Clathrin effect on rCARDS toxin-mediated vacuole formation. S-siRNA-treated (i) and C-siRNA-treated (ii) HeLa cells were incubated with 50 µg/ml of rCARDS toxin for 24 h at 37°C and analyzed under light microscopy for vacuole formation as described previously [14].

To further confirm that CARDS toxin entry requires clathrin-mediated endocytosis, we used clathrin heavy chain siRNA (C-siRNA) to selectively reduce the expression of clathrin in HeLa cells. After two successive transfections of C-siRNA into HeLa cells, clathrin levels were down regulated by 91% as compared with cells transfected with scrambled siRNA (S-siRNA) under similar conditions (Fig. 5A–B). HeLa cells transfected with C-siRNA and S-siRNA were analyzed for rCARDS toxin internalization by confocal laser scanning microscopy and Biotin-CARDS internalization. Reduction in the amount of clathrin heavy chain in C-siRNA transfected HeLa cells decreased internalization of Biotin-CARDS toxin by 88% but did not interfere with toxin binding to HeLa cell surfaces (Fig. 5C–D). S-siRNA-transfected HeLa cells had no effect on binding and internalization of CARDS toxin (Fig. S3 and Fig. 5C–D). Confocal laser scanning microscopy further confirmed inhibition of uptake of rCARDS toxin and transferrin in C-siRNA transfected HeLa cells (Fig. S3). Also, rCARDS toxin-treated C-siRNA-transfected HeLa cells did not develop vacuoles, which is consistent with the reduction in uptake of rCARDS toxin. In contrast, S-siRNA transfected HeLa cells exhibited the toxin-mediated, ‘normal’ vacuolated cell phenotype (Fig. 5E).

## Discussion

Using fluorescent- and biotin-tagged rCARDS toxin, we showed that rCARDS toxin bound to mammalian cells in a concentration-dependent manner, followed by rapid internalization, which was temperature dependent and mediated by clathrin-associated endocytosis. Recently, we reported that CARDS toxin exhibits ADP-ribosylating transferase activity and induces vacuolization in mammalian cells [11], [14], tracheal organ cultures [11] and animals [12]; the latter recapitulates the vacuolization phenotype and loss of tissue integrity that accompanies *M. pneumoniae* infection [27], [28]. Interestingly, CARDS toxin initiates proinflammatory responses that also mimic *M. pneumoniae*-induced pathologies in animal models [8], [12]. Furthermore, the high levels of CARDS toxin expression observed during *M. pneumoniae* infection in animal models and, importantly, the detection of CARDS toxin in the lungs and bronchoalveolar fluid of infected humans establish this protein as a bona fide virulence factor [12], [15], [16], [17], [18].

Bacterial ADP-ribosylating toxins interact with receptors on host cell surfaces [20]. Consistent with this property, the internalization of rCARDS toxin that followed its binding to mammalian cells required a temperature shift from 4°C to 37°C, reinforcing active receptor-mediated uptake [29], [30]. For example, internalization of Biotin-CARDS toxin was minimal at 4°C, while as much as 90% of surface-bound toxin was internalized at 37°C within 1 h in HeLa cells (Fig. 2B) and 86% of surface-bound toxin was internalized in A549 cells (data not shown). Also, the ability of rCARDS toxin to bind and enter a wide range of mammalian cell lines and exhibit biological activity [14] suggests that rCARDS toxin binds to common surface receptor(s), unlike diphtheria toxin, but similar to other bacterial toxins [31], [32]. Interestingly, we initially identified CARDS toxin as a surfactant protein–A (SP-A) binding protein [13] but our studies (Fig. S1) revealed that binding and internalization of CARDS toxin can occur in cells deficient in SP-A, suggesting that alternative receptors exist; this is consistent with the multi-tissue pathologies associated with *M. pneumoniae* infections and the fact that other toxins bind to multiple surface membrane receptors [33]. In the current study we focused on surface binding and internalization of rCARDS toxin via clathrin-mediated mechanisms, which does not preclude multiple and redundant receptor-mediated pathways that facilitate the binding and entry of CARDS toxin at airway and extrapulmonary sites.

The uptake mechanisms associated with most bacterial toxins start with clathrin-coated pits or clathrin-independent endocytosis pathways [23], [24], [34]. Clathrin-independent endocytosis includes an array of endocytic mechanisms including caveolar or non-caveolar lipid rafts, macropinocytsis and phagocytosis [35]. By using pharmacological drugs that inhibit endocytosis through either clathrin- or caveolae-mediated pathways, we found that rCARDS toxin is endocytosed by clathrin-coated vesicles. Filipin and MβCD which block caveolar-mediated endocytosis did not inhibit CARDS toxin internalization. However MDC, a clathrin-coated, vesicle-mediated inhibitor markedly reduced CARDS toxin uptake. Many bacterial toxins including Pseudomonas exotoxin-A [36] and Shiga toxin [37], [38] are endocytosed through clathrin-coated vesicles and clathrin-independent pathways [24], [25], [26]. Interestingly, cholera toxin is mainly endocytosed by a caveolae-dependent mechanism [39] while diphtheria toxin is limited to clathrin-dependent endocytosis [40].

To further confirm the role of clathrin in rCARDS toxin uptake, we employed siRNA technology to deplete clathrin heavy chain and analyzed rCARDS toxin internalization. The siRNA technology for silencing clathrin has been used successfully to study endocytic accessory proteins and receptor trafficking in mammalian cells [41], [42]. Following the depletion of clathrin protein by 91% using C-siRNA, 88% of Biotin-CARDS toxin internalization was inhibited. Confocal microscopy of siRNA clathrin-treated cells also showed marked reductions in both clathrin protein and toxin uptake. These data indicate that uptake of CARDS toxin in mammalian cells is similar to the mechanisms used by many clathrin-dependent endocytosed toxins [43]. Therefore, using a range of methodologies, we identified clathrin-mediated endocytosis as the main pathway for CARDS toxin internalization. Identification of functional host cell surface receptors that enable CARDS toxin binding and internalization will help us understand the mechanisms by which CARDS toxin elicits cytopathology, thus offering opportunities for the design of rational neutralizing strategies to prevent CARDS toxin-mediated inflammation and tissue injury.

## Materials and Methods

### Cell Culture, Proteins, Antibodies and Chemicals

Mammalian cells of different origin were cultured in their respective media (cell culture media and cell lines were obtained from ATCC): HeLa (CCL-2), HEp2 (CCL-23), and Vero (CCL-81) cells were cultured in Minimal Essential Medium (MEM); A549 (CCL-185) and CHO-K1 (CCL-61) were grown in F12-K medium. All media contained 10% fetal bovine serum (FBS) (Atlas Biologicals), penicillin (100 units/ml), and streptomycin (100 µg/ml) and cell cultures were passaged in 5% CO_2_, 95% humidified condition at 37°C. rCARDS toxin and rabbit polyclonal anti-CARDS toxin antibodies were used as described previously [11]. Human holo-transferrin (Tf) was purchased from Sigma-Aldrich. Cholera toxin was obtained from List Biological Laboratory. Rabbit polyclonal anti-clathrin and anti-cholera antibodies were obtained from Abcam, and anti-GAPDH rabbit monoclonal antibodies were from Cell Signaling. Alexa Fluor conjugated secondary antibodies were from Invitrogen Corp., USA, and other biochemical were purchased from Sigma-Aldrich.

### Immunofluorescence Staining and Confocal Laser Scanning Microscopy

Mammalian cells (0.5×10^5^cells/well) were grown in appropriate culture medium on cover glasses at 37°C. For toxin binding studies, cells were washed and incubated with rCARDS toxin (10 µg/ml) in serum-free culture medium at 4°C for the indicated time period. Cell monolayers were washed three times with PHEM buffer (60 mM PIPES, 25 mM HEPES, 2 mM EGTA and 10 mM MgCl_2_ pH7.2), fixed with 2% paraformaldehyde for 20 min, permeabilized with 0.1% Triton X-100 for 10 min and blocked with 5% normal goat serum in PHEM buffer. Then, cells were incubated with rabbit polyclonal anti-CARDS toxin antibodies (1∶1000 dilution) in 0.5% normal goat serum in PHEM buffer for 1 h, washed three times with 0.5% normal goat serum in PHEM buffer and further incubated with secondary goat polyclonal anti-rabbit antibodies (1∶500 dilution) labeled with Alexa Fluor 633 (Invitrogen) for 1 h. Test samples were washed with PHEM buffer and mounted on glass slides using Vectashield hard fix mounting medium containing DAPI (4′,6-diamidino-2-phenylindole dihydrochloride) stain (Vector Lab). For toxin internalization studies, HeLa cells were incubated with 10 µg rCARDS toxin at 37°C for 1 h. After removing unbound toxin by washing cells three times in serum-free medium, cells were incubated in complete culture medium at 37°C. At specific intervals, test samples were processed as indicated above for binding and internalization studies. Confocal microscopic analysis was performed using an Olympus XI-81 confocal laser scanning microscope with Flow view 1000 imaging software. Z series at 0.5 micrometer sections were obtained by combining a series of x-y scans taken along the z axis. Cells without CARDS toxin were processed similarly to serve as negative controls. In parallel, cells treated with cholera toxin (0.5 µg) or Tf (10 µg), plus their respective antibodies at 1∶500 dilution, served as positive controls for caveolin-mediated and clathrin-mediated endocytosis.

### Quantification of Cell Surface Binding of rCARDS Toxin

To study rCARDS toxin binding to cell surfaces, we labeled toxin with DyLight 649 fluorescence dye (DL649-CARDS) according to the manufacturer’s protocol (Thermo Scientific). HeLa cells grown in 96-well culture plates (5×10^4^ cells/well) were incubated with varying concentrations (0.1 to 25 µg/ml) of DL649-CARDS toxin for 1 h at 4°C. To determine time course binding kinetics, we added 10 µg/ml of DL649-CARDS toxin to cell monolayers for 15 min to 8 h at 4°C. Unbound toxin was removed by washing cells in cold PBS buffer, and toxin binding was measured by fluorescence. For competitive binding assays, HeLa cells were pre-incubated with or without 10 µg of unlabeled CARDS toxin for 10 min at 4°C, prior to addition of 1 µg of DL649-CARDS toxin. Incubation was continued for 1 h at 4°C, and binding was analyzed by fluorometry using 625 nm-red filter (Turner Biosystem).

Time-dependent rCARDS toxin binding was examined using pacific blue-A fluorescence dye labeled rCARDS (PBA-CARDS) toxin labeled according to manufacturer protocol (Thermo Scientific). One million HeLa cells were incubated with 10 µg/ml of PBA-CARDS toxin at 4°C for 5 min to 8 h on a rocking shaker. Unbound toxin was washed three times with cold PBS and removed by centrifugation at 2000×g at 4°C for 5 min, and the amount of bound toxin was analyzed by flow cytometry (UTHSCSA core flow cytometry facility; FACS Caliber system). Cells were analyzed using forward scatter and side scatter gates to include all fluorescing individual cells at 405 nm channel. Cells treated identically without PBA-CARDS toxin served as negative controls. In parallel studies, HeLa cells were incubated with 10 µg/ml of rCARDS toxin as above and treated with rabbit polyclonal anti-CARDS toxin antibodies (1∶1000) in PBS with 3% fetal bovine serum (FBS) for 1 h at 4°C. After three washes with PBS-FBS, cell preparations were incubated with Alexa-Fluor 488 conjugated goat anti-rabbit secondary antibodies (1∶500) (Invitrogen) for 30 min at 4°C. rCARDS toxin-bound HeLa cells were analyzed using forward scatter and side scatter gates to include all individual cells stained with Alexa Fluor 488. Cells treated similarly without rCARDS toxin served as negative controls.

### Internalization of rCARDS Toxin

To measure endocytosed toxin quantitatively, we labeled rCARDS toxin with biotin (Biotin-CARDS) according to the manufacturer’s instructions, using EZ-Link sulfo-*N*-hydoroxylsulfosuccinimide-biotin (sulfo-NHS-SS-biotin) (Pierce). HeLa or A549 cells were grown in 96-well culture plates (5×10^4^ cells/well) and washed three times with cold Hanks’ balanced salt solution containing 0.1% bovine serum albumin (HBSS-BSA). Biotin-CARDS toxin (1–10 µg) in HBSS was added to individual wells at 4°C and at specific time intervals (15 min to 8 h) cell preparations were washed with ice cold HBSS three times to remove unbound toxin. Then, cells were fixed for 20 min with 0.25% glutaraldehyde in PBS buffer and blocked with 3% BSA in PBS for 1 h. To detect surface bound Biotin-CARDS toxin, cells were incubated for 1 h with horseradish peroxidase (HRP)-conjugated streptavidin (Pierce); reactions were stopped with 1 M sulfuric acid-stop solution (Thermo Scientific); and developed color was determined after 20 min incubation with BM blue substrate (Roche Diagnostic) at 450 nm using ELISA reader (MRX Dynatech Lab). Mock cells with no Biotin-CARDS toxin added provided background values.

To assess CARDS toxin internalization, HeLa cells were treated with Biotin-CARDS toxin (10 µg) in HBSS per well at 4°C for 30 min. Excess rCARDS toxin was removed by washing with cold HBSS, and cell monolayers were rapidly brought to 37°C with warmed culture medium. At each time interval (5 min to 1 h), reactions were quickly stopped by returning monolayers to 4°C in cold HBSS. Cell preparations were incubated with the membrane impermeant reducing agent, 2-mercaptoethanesulfonic acid (MESNA; 0.5 M) for 30 min at room temperature to remove biotin from cell surface-bound toxin molecules. Cell preparations were fixed with glutaraldehyde in PBS, permeablized with 0.1% Triton X-100 in PBS for 5 min, and blocked with 3% BSA. Internalized rCARDS toxin was detected using HRP conjugated streptavidin followed by BM blue substrate as described above. HeLa cells treated similarly with Biotin-CARDS for 30 min at 4°C along with MESNA, but without temperature shift, served as negative controls to check for biotin removal efficiency of MESNA. Toxin-treated cells not incubated with MESNA served as positive controls to quantify the amount of total bound and internalized toxin. Cells treated similarly but with no toxin added served as background controls. The percentage of HeLa and A549 cells that internalized CARDS toxin was calculated by dividing the internalized CARDS toxin signal by the total bound signal.

### Pharmacological Drug Treatments

HeLa or A549 cell monolayers were pre-incubated for 30 min at 37°C in fresh culture medium containing one of the following inhibitors: monodansylcadaverine (100 µM), methyl-β-cyclodextrin (5 mM) and filipin (1 µg/ml). After these treatments, rCARDS toxin (10 µg/ml) or Tf (10 µg/ml) or cholera toxin (0.5 µg/ml) was added for 1 h. Cells were washed three times in PHEM buffer, and binding and internalization of CARDS toxin were analyzed by immunofluorescence staining as described above.

### Clathrin Silencing by Using RNA Interference

HeLa cells grown to 70% confluence were transfected for 4 h with Fugene 6 (Roche) containing a mixture of 100 pMol synthetic double-stranded small interfering RNA (siRNA) of clathrin heavy chain (CHC) or scrambled siRNA (negative control) (Santa Cruz Biotechnology). After 72 h of transfection, a second siRNA transfection was performed and 24 h after the second transfection, transfected and non-transfected control cells were treated with 10 µg/well of rCARDS toxin for 1 h. Cells were washed three times in PHEM buffer, and binding and internalization of rCARDS toxin were analyzed by immunofluorescence staining. For immunoblotting, proteins were resolved on 4–12% Nu PAGE and transferred to nitrocellulose membrane. To quantify the relative expression levels of clathrin compared to glyceraldehyde 3-phosphate dehydrogenase (GAPDH; control), individual membranes were probed with rabbit polyclonal anti-clathrin antibodies (Abcam; 1∶500) or rabbit monoclonal anti-GAPDH antibodies (Cell Signaling Co., 1∶2000) followed by HRP-conjugated goat anti-rabbit IgG (Cell Signaling; 1∶2000 dilution) and visualized by chemiluminescence. Protein band intensities from autoradiograms were quantified using Kodak 1D gel analysis software. For vacuolization experiments, normal and siRNA transfected HeLa cells were treated for 24-to-72 h with 10 µg of rCARDS toxin as described earlier and analyzed by light microscopy for vacuolization.

## Supporting Information

Figure S1 **rCARDS toxin binds to and is internalized by a wide range of cell lines.** HEp2, A549, Vero and CHO mammalian cells were treated with 10 µg/ml of rCARDS toxin for 1 h at 37°C. Cells were fixed, permeabilized and analyzed for binding and internalization of rCARDS toxin using rabbit polyclonal anti-CARDS toxin antibodies and confocal laser scanning microscopy as described under Fig. 1 legend.
(TIF)

Figure S2 **Effects of different inhibitory compounds on clathrin-mediated cell entry of rCARDS toxin.** HeLa cells cultured on cover glass were pre-incubated 30 min at 37°C with 5 mM of Methyl-2-cyclodextrin or 100 µM of Monodansylcadaverine or 1 µg Filipin. Subsequently, cells were treated with 10 µg/ml rCARDS toxin or 10 µg/ml Tf or 0.5 µg/ml of cholera toxin. Treated cells were processed for immunofluorescence as described in Materials and Methods. Images were obtained by confocal laser scanning microscopy. Tf and cholera toxin served as positive controls for clathrin-mediated endocytosis and caveolin-mediated endocytosis, respectively.
(TIF)

Figure S3 **Effect of clathrin depletion on rCARDS toxin uptake by HeLa cells.** C-siRNA or S-siRNA transfected HeLa cells were incubated with 10 µg/ml of rCARDS toxin (A–B) or 10 µg/ml of Tf (C-D) at 37°C. Immunofluorescence labeling was performed using rabbit polyclonal anti-CARDS toxin antibodies (1∶1000) or anti-Tf mouse antibodies (1∶500) along with corresponding secondary antibodies tagged with Alex-Flour 633 (Red). Nuclei were labeled with DAPI (Blue). All images of 0.5 micrometer z-section and cross sectional views were obtained using confocal laser scanning microscope.
(TIF)
