# Supplementary figures and images for: Mycoplasma pneumoniae CARDS Toxin Is Internalized via Clathrin-Mediated Endocytosis
Source: PLoS One. 2013 May 7;8(5):e62706. doi: 10.1371/journal.pone.0062706 (PMC3647021; doi:10.1371/journal.pone.0062706)

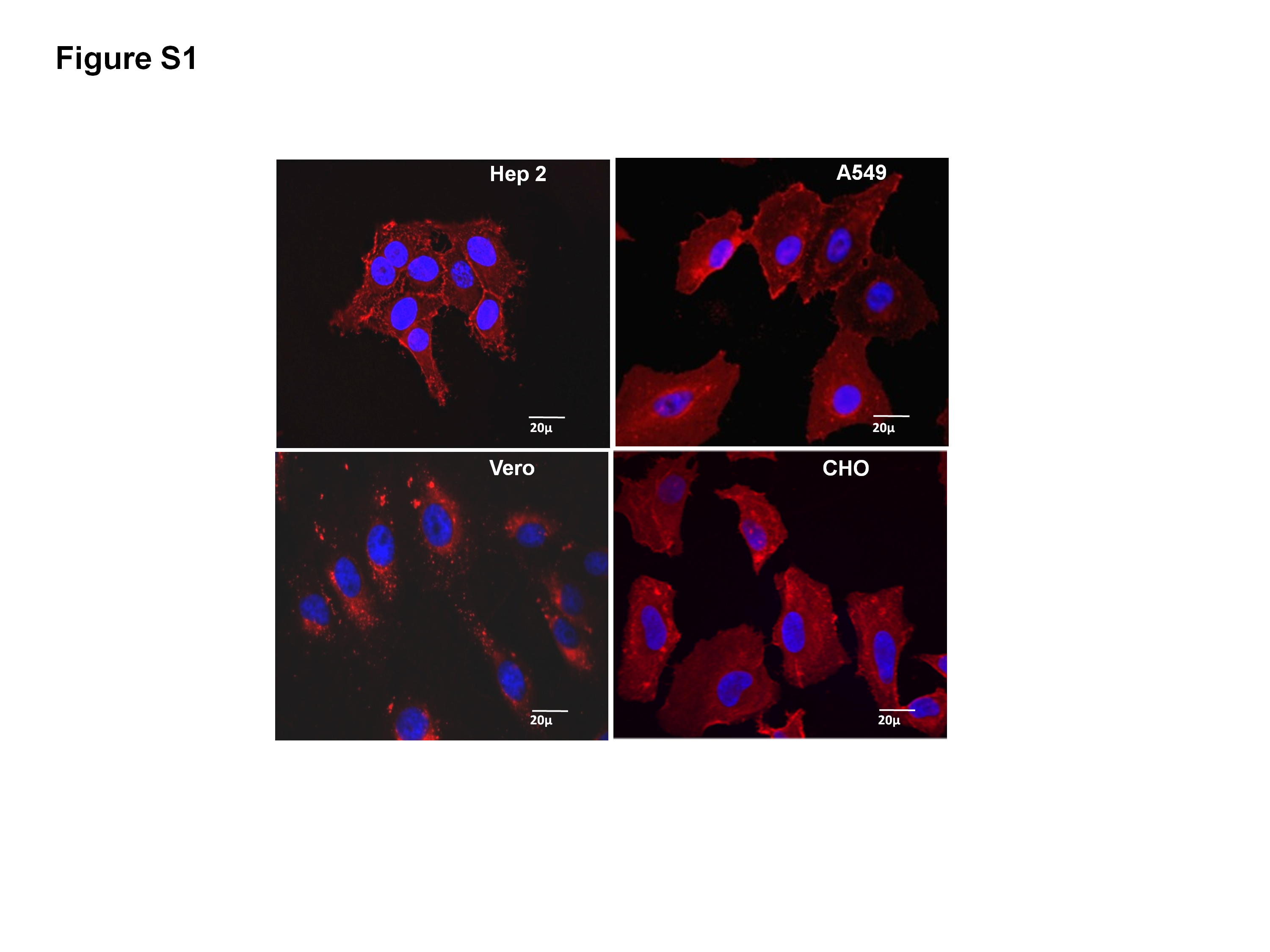

Supplement: Figure S1 — rCARDS toxin binds to and is internalized by a wide range of cell lines. HEp2, A549, Vero and CHO mammalian cells were treated with 10 µg/ml of rCARDS toxin for 1 h at 37°C. Cells were fixed, permeabilized and analyzed for binding and internalization of rCARDS toxin using rabbit polyclonal anti-CARDS toxin antibodies and confocal laser scanning microscopy as described under Fig. 1 legend. (TIF) [file pone.0062706.s001.tif]

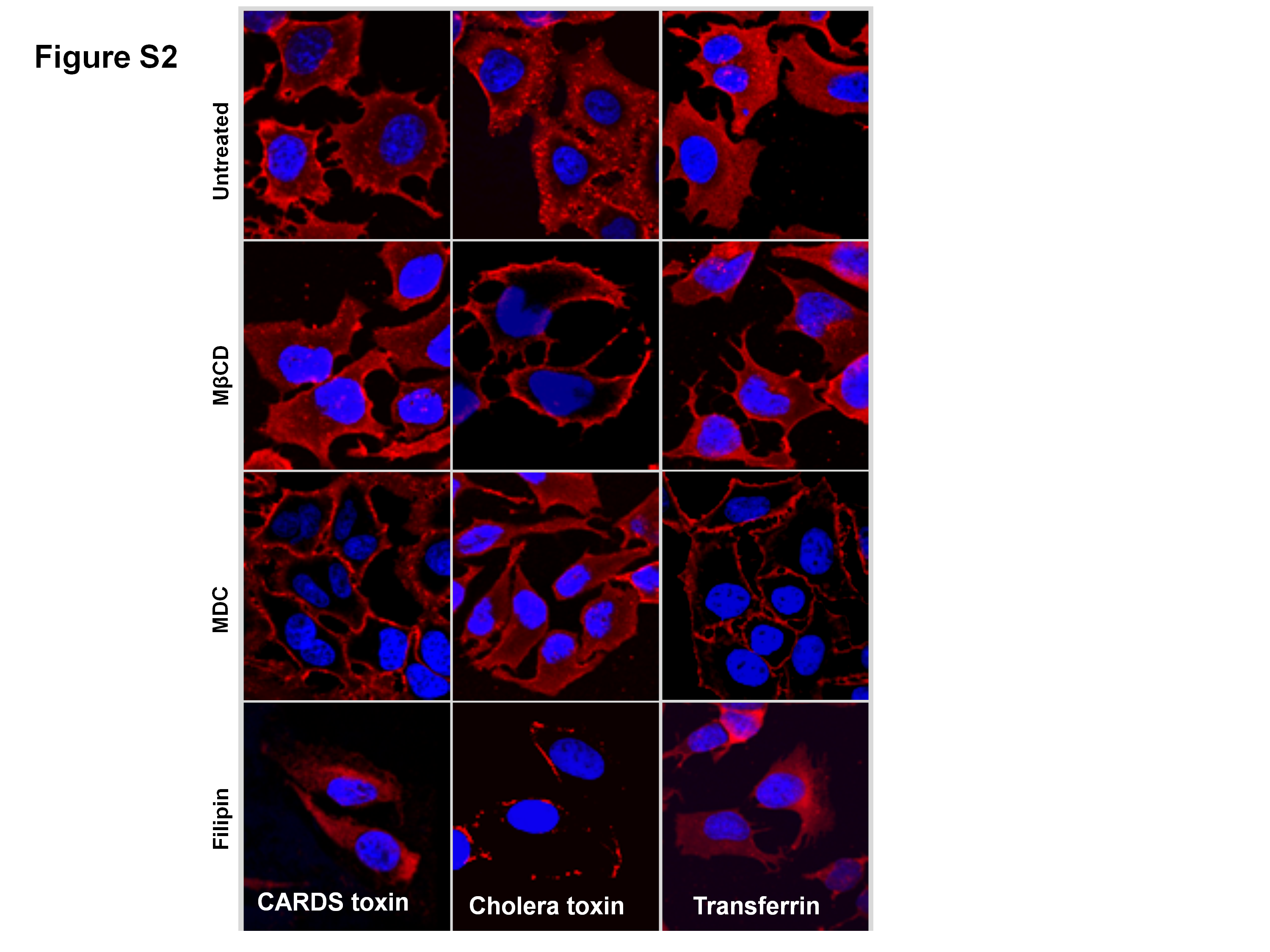

Supplement: Figure S2 — Effects of different inhibitory compounds on clathrin-mediated cell entry of rCARDS toxin. HeLa cells cultured on cover glass were pre-incubated 30 min at 37°C with 5 mM of Methyl-2-cyclodextrin or 100 µM of Monodansylcadaverine or 1 µg Filipin. Subsequently, cells were treated with 10 µg/ml rCARDS toxin or 10 µg/ml Tf or 0.5 µg/ml of cholera toxin. Treated cells were processed for immunofluorescence as described in Materials and Methods. Images were obtained by confocal laser scanning microscopy. Tf and cholera toxin served as positive controls for clathrin-mediated endocytosis and caveolin-mediated endocytosis, respectively. (TIF) [file pone.0062706.s002.tif]

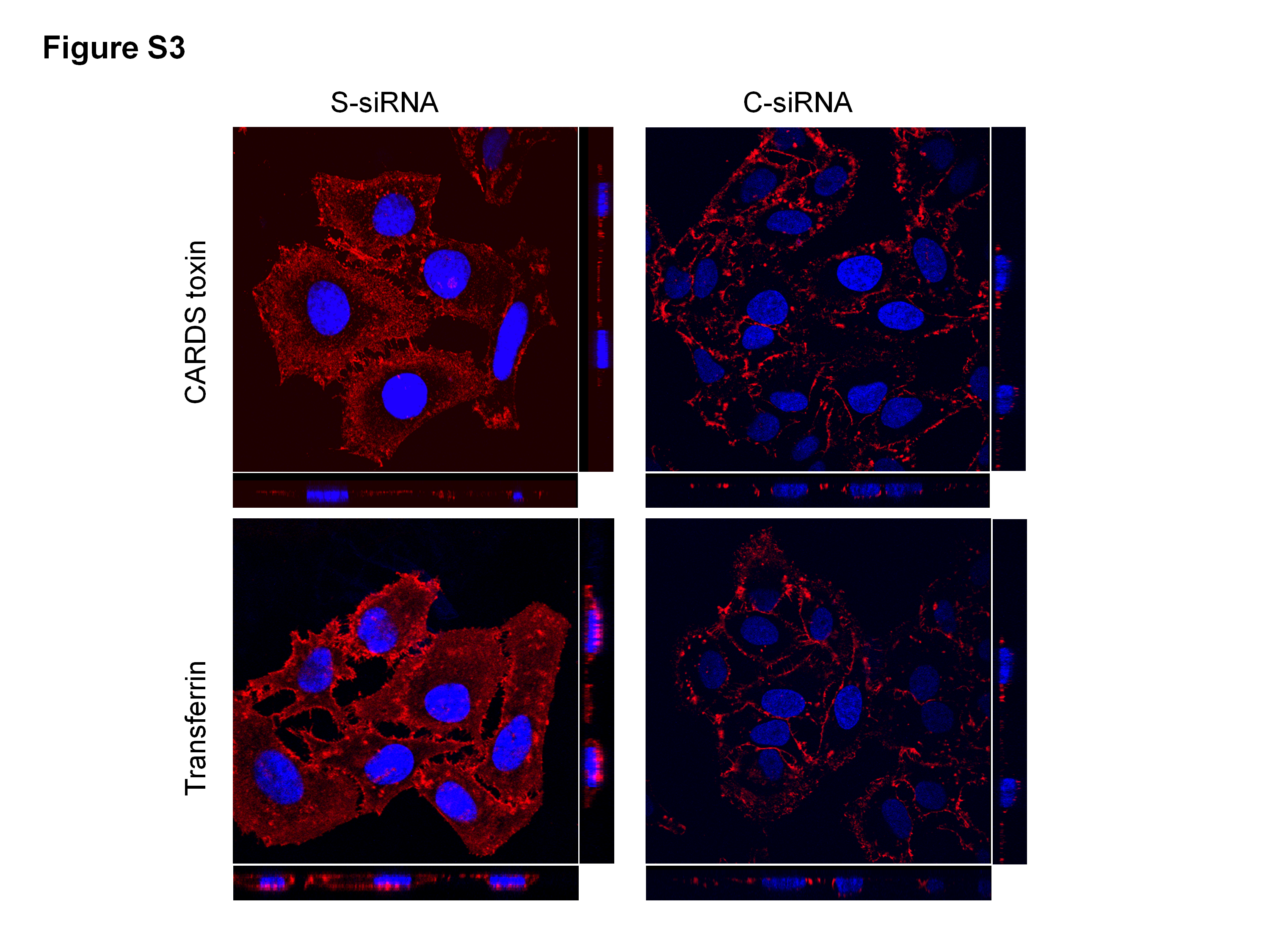

Supplement: Figure S3 — Effect of clathrin depletion on rCARDS toxin uptake by HeLa cells. C-siRNA or S-siRNA transfected HeLa cells were incubated with 10 µg/ml of rCARDS toxin (A–B) or 10 µg/ml of Tf (C-D) at 37°C. Immunofluorescence labeling was performed using rabbit polyclonal anti-CARDS toxin antibodies (1∶1000) or anti-Tf mouse antibodies (1∶500) along with corresponding secondary antibodies tagged with Alex-Flour 633 (Red). Nuclei were labeled with DAPI (Blue). All images of 0.5 micrometer z-section and cross sectional views were obtained using confocal laser scanning microscope. (TIF) [file pone.0062706.s003.tif]
